# A phase II clinical study of 13‐deoxy, 5‐iminodoxorubicin (GPX‐150) with metastatic and unresectable soft tissue sarcoma

**DOI:** 10.1002/cam4.2136

**Published:** 2019-04-23

**Authors:** Brian A. Van Tine, Mark Agulnik, Richard D. Olson, Gerald M. Walsh, Arthur Klausner, Nicole E. Frank, Todd T. Talley, Mohammed M. Milhem

**Affiliations:** ^1^ Division of Medical Oncology Washington University in St. Louis St Louis Missouri; ^2^ Division of Hematology/Oncology, Feinberg School of Medicine Northwestern University Chicago Illinois; ^3^ Gem Pharmaceuticals LLC Birmingham Alabama; ^4^ College of Pharmacy Idaho State University Meridian Idaho; ^5^ Division of Hematology/Oncology University of Iowa Hospitals and Clinics Iowa City Iowa

**Keywords:** anthracyclines, cardiotoxicity, doxorubicin, GPX‐150, phase II, soft tissue sarcoma

## Abstract

**Background:**

13‐Deoxy, 5‐iminodoxorubicin (GPX‐150) is a doxorubicin (DOX) analog synthesized to reduce the formation of reactive oxygen species and the cardiotoxic metabolite, doxorubiciniol, the two pathways that are linked to the irreversible, cumulative dose‐dependent cardiotoxicity of DOX. In a preclinical chronic models and a phase I clinical study of GPX‐150, no irreversible, cumulative dose‐dependent cardiotoxicity was demonstrated. Recent studies suggest that DOX cardiotoxicity may be mediated, at least in part, by the poisoning of topoisomerase IIβ.

**Patients and Methods:**

An open‐label, single‐arm phase II clinical study in metastatic and unresectable soft tissue sarcoma (STS) patients was initiated to further evaluate the efficacy and safety of GPX‐150, including cardiac function, specifically left ventricular ejection fraction (LVEF).

**Results:**

GPX‐150 was administered at 265 mg/m^2^ every 3 weeks for up to 16 doses with prophylactic G‐CSF until progression, death, or patient withdrawal from the study. GPX‐150 exhibited efficacy assessed as progression‐free survival (PFS) rates of 38% and 12% at 6 and 12 months and an overall survival rate of 74% and 45% at 6 and 12 months. GPX‐150–treated patients did not develop any evidence of irreversible, cumulative dose‐dependent chronic cardiotoxicity. Toxicities included grade 3 anemia, neutropenia, and one grade 4 leukopenia. Correlative analysis demonstrated that GPX‐150 was more selective than DOX for the inhibition of topoisomerase IIα over IIβ in vitro.

**Conclusion:**

These results suggest future studies are warranted to further evaluate the clinical efficacy of GPX‐150 in STS, perhaps at doses higher than 265 mg/m^2^.

## 1 INTRODUCTION

Doxorubicin (DOX) as a single agent or in combination with ifosfamide has been the mainstay of first‐line treatment for advanced or metastatic soft tissue sarcoma (STS) for more than 40 years.^1^, ^2^, ^3^, ^4^ Recently, DOX in combination with olaratumab, a monoclonal antibody against platelet‐derived growth factor receptor alpha, demonstrated superiority over DOX alone and was granted accelerated approval by the FDA in 2016 as the first‐line therapy for metastatic STS.^5^ This was the first substantial change in chemotherapy for first‐line treatment of STS and solidifies the role of continued DOX use in STS. The continued role of DOX in STS and other cancers justifies the rationale for developing a safer and less cardiotoxic DOX analog. DOX can induce a chronic, irreversible cumulative cardiotoxicity that occurs in approximately 10% of patients, which limits its clinical utility.^6^ The irreversible cardiotoxicity is dependent on the cumulative dose, which limits cumulative DOX use below a dose of 350‐500 mg/m^2^. Even with early intervention with Angiotensin‐converting enzyme (ACE) inhibitors and beta blockers, not all patients recover within 5 years from the cardiotoxicity that develops after completion of DOX therapy.^6^ Cardiotoxicity also impairs the clinical use of DOX in combination with other chemotherapeutic agents such as paclitaxel and trastuzumab, even when the combination improves the clinical outcome.^7^, ^8^, ^9^, ^10^, ^11^, ^12^

The predominant theory for irreversible, cumulative DOX cardiotoxicity has evolved over many years and is believed to result from a quinone‐derived reactive oxygen species (ROS) and the formation of the cardiotoxic metabolite, doxorubicinol, in cardiac myocytes.^13^, ^14^, ^15^, ^16^, ^17^, ^18^ More recently, an additional mechanism has been purported to contribute to chronic DOX cardiotoxicity related to inhibition of topoisomerase IIβ that is expressed in the adult heart.^19^ GPX‐150 was specifically engineered to reduce or prevent cardiac ROS and doxorubicinol formation. In addition, GPX‐150 has been recently shown to spare inhibition of human topoisomerase IIβ in isolated in vitro decatenation assays.^20^

Preclinical studies demonstrated that GPX‐150 did not cause a chronic irreversible cardiotoxicity and the pharmacokinetics and cardiac effects of GPX‐150 were evaluated in a phase I clinical trial.^20^, ^21^ This phase I clinical trial was a dose‐escalation study designed to determine the pharmacokinetics and maximum tolerated dose (MTD) of GPX‐150. The results from the trial demonstrated no cardiotoxicity in 24 patients, including four patients who had been previously treated with anthracyclines. The MTD was determined by neutropenia in the absence of granulocyte colony stimulating factors. GPX‐150 demonstrated stable disease in five out of seven patients, including three patients with STS. This provided the rationale for a phase II single‐arm, open clinical trial with GPX‐150 in patients with STS to evaluate progression‐free rate (PFS) with comparison to historical DOX PFS in STS. In addition, this provided the opportunity to evaluate the safety, including that of irreversible, cumulative cardiotoxicity, of up to 16 cycles of GPX‐150 administered every 3 weeks at the MTD defined in the phase I clinical trial.

## 2 PATIENTS AND METHODS

### 2.1 Patients

The study population consisted of patients with metastatic or locally advanced unresectable STS. Individuals (age ≥18 years) with histological documentation reviewed by institutional sarcoma pathologists at their treating institutions of advanced and/or metastatic STS of intermediate or high histologic grade for which an anthracycline was an appropriate therapy and who gave informed written consent according to Food and Drug Administration and institutional guidelines were eligible. The patients could not have received prior chemotherapy for their current sarcoma, except for gemcitabine and/or docetaxel as adjuvant therapy completed at least 6 months prior to the first planned dose of GPX‐150. Patients with the following sarcoma subtypes were excluded from the study: well‐differentiated liposarcoma or atypical lipomatous tumor, embryonal or alveolar rhabdomyosarcoma, Ewing's sarcoma, gastrointestinal stromal tumor (GIST), dermatofibrosarcoma protuberans, alveolar soft part sarcoma, solitary fibrous tumor, clear cell sarcoma, kaposi sarcoma, extraskeletal myxoid chondrosarcoma, PEComa (perivascular epithelial cell tumor), myoepithelioma/mixed tumor, sarcomas arising from bone or cartilage (chondrosarcoma, osteosarcoma, chordoma), or any patient that was eligible for curative intent therapy. Patients who underwent radiotherapy to greater than 25% of bone marrow volume were also excluded from the study.

Other inclusion criterion included disease that was measurable using RECIST 1.1 and an ECOG performance status of 0‐2. Patients were also required to have adequate cardiac function with left ventricular ejection fraction (LVEF) above the institution's lower limit of normal and QTcF ≤450 msec for males or 470 msec for females. Patients also agreed to use a highly reliable method of birth control for the duration of the study and women of childbearing potential had a serum pregnancy test performed within 28 days prior to the first day of GPX‐150 dosing.

Patients were required to have adequate bone marrow, liver, and renal function (absolute neutrophil count [ANC] >1500/mm^3^, platelet count > 100 000/mm^3^, total bilirubin <1.5 × ULN, ALT or AST < 2.5 × ULN [upper limit of normal; for subjects with documented liver metastases, ALT and AST < 5 × ULN], serum creatinine < 1.5 x ULN, international normalized ratio [INR] or activated partial thromboplastin time [PTT] ≤1.5 × ULN, if not therapeutically anticoagulated and serum albumin > 3.0 gm/dL). Additionally, eligible patients could not have congestive heart failure greater than Class II New York Heart Association functional classification, pericarditis, myocardial infarction within 6 months, or symptomatic coronary artery disease, active infection requiring systemic antibacterial/antibiotic, antifungal, or antiviral therapy or documented metastases to the brain or meninges. Other criteria for patient exclusion were any malignancy other than STS within the last 5 years prior to screening, with the exception of cervical carcinoma in situ, basal cell carcinoma, or superficial bladder tumors that were successfully and curatively treated with no evidence of recurrent or residual disease, currently pregnant or nursing women or known allergy to any of the study drugs or their excipients.

### 2.2 Study design

The primary objective was to determine the efficacy of GPX‐150 administered intravenously once every 3 weeks to patients with STS as determined by progression‐free rate (PFR) at 12 months of treatment and to describe the safety profile of GPX‐150. The secondary objective was to describe the effects of GPX‐150 on STS as assessed by secondary efficacy measures: (a) PFR at 6 months, (b) progression‐free survival (PFS, defined as time to disease progression or death, (c) time to progression (TTP), (d) overall survival (OS), defined as time to death, (e) tumor response defined as complete response (CR), partial response (PR), or stable disease (SD), (f) duration of response, (g) duration of overall response (CR or PR), (h) best response during study, and (i) duration of best response.

### 2.3 Drug formulation and administration

GPX‐150 for injection was supplied as a lyophilized powder formulation in sterile 50‐mL amber glass vials containing 50 mg of the drug (±15%) and 250 mg of lactose monohydrate. The actual amount of GPX‐150 for injection per vial was measured and reported on a certificate of analysis (COA) prepared for each manufacturing lot. Pharmacists were instructed to refer to the COA accompanying each shipment of GPX‐150 for the amount of investigational product in each vial in order to calculate dose volumes. Vials were protected from light and stored between −25° and −10°C (−13° and 14°F). The lyophilized powder was reconstituted by the addition to the vial of 40 mL of 0.9% sodium chloride injection. The vial was shaken and the contents were completely dissolved after a period of 30 minutes at room temperature. Sites were instructed that the reconstituted product should be used promptly 30 minutes after reconstitution and no more than 22 hours following reconstitution.

GPX‐150 was administered at a starting dose of 265 mg/m^2^. Sequential dose reductions to 200 mg/m^2^ and then to 150 mg/m^2^ were allowed based on predetermined guidelines. The undiluted calculated dose was added to an intravenous (IV) bag for administration via a constant infusion pump at an infusion rate of 2 mL/min through IV tubing. The IV tubing was flushed with an infusion of 0.9% sodium chloride for injection. The IV tubing was attached to a central line or angiocath inserted into a large vein. A dose of GPX‐150 was administered every 21 days (1 cycle). Treatment continued for 16 cycles, or until radiographically confirmed disease progression, death, or unacceptable toxicity.

Based on the phase I clinical study, it was expected that the dose limiting toxicity of GPX‐150 is myelosuppression. Therefore, G‐CSF was used in all subjects. Primary prophylaxis with G‐CSF was initiated after dose 1 and prophylaxis or treatment was administrated by the investigators according to ASCO guidelines.^22^

A continuation of dosing (compassionate use) past one year was allowed. Prior to initiation of the first post‐cycle 16 dose, all end of study radiographic studies and tumor assessments were performed before the initiation of continued dosing. Radiological studies and tumor assessments were performed every 9 weeks during the dosing continuation period. Patients continued to receive doses of GPX‐150 every 3 weeks for a maximum of eight additional cycles or until radiographically demonstrated disease progression, unacceptable toxicity, or withdrawal of consent by the patient. Dosing could also be terminated before the maximum of eight cycles if there was an insufficient supply of GPX‐150. Upon completion of the dosing continuation period, all end of study final visit assessments were performed.

### 2.4 Assessment of efficacy and adverse effects

The incidence, nature, and severity of adverse events (AEs) were determined according to the National Cancer Institute Common Terminology Criteria for Adverse Events (NCI:CTCAE) version 4.

### 2.5 Statistics and IRB approvals

Data were summarized using descriptive statistics (number of subjects [n], mean, median, standard deviation, minimum, and maximum) for continuous variables. Categorical (discrete) variables were summarized using frequencies and percentages using MedCalc Version 18 (MedCalc Software bvba, Ostend, Belguim; http://medcalc.org;2018). Time to event variables were summarized using the number observed, number censored, median, and 25th and 75th percentiles from Kaplan‐Meier (KM) curves. Data were summarized using descriptive statistics (number of subjects [n], mean, median, standard deviation, minimum, and maximum) for continuous variables. Categorical (discrete) variables were summarized using frequencies and percentages. Mean values for QRS complex duration, QT interval, and QTcF were compared at screening and at final visit using Student's unpaired *t* test (significance level was *P* < 0.05). LVEF mean values at screening and at final visit were compared using Student's unpaired *t* test (*P* < 0.05 was used as the level of significance).The trial was an open‐label single‐arm phase II study at three University sites, University of Iowa (Iowa City, IA) Northern University (Chicago, IL), and Washington University in Saint Louis, (St. Louis, MO), and was approved by their corresponding Institutional Review Boards. An informed consent was obtained from all individual participants included in the study. The trial was registered with ClinicalTrials.gov (Identifier No. NCT02267083).

### 2.6 Correlative studies

#### 2.6.1 Human topoisomerase IIα and β assays

Four units of human topoisomerase IIα or IIβ (Lae Biotech International, Rockville, MD) were incubated for 60 minutes at room temperature in the presence of assay buffer (10 mmol/L Tris‐HCl, pH 8.0, 50 mmol/L NaCl, 0.1 mmol/L EDTA, 50 mmol/L KCl, 5 mmol/L MgCl_2_, 15 µg/mL BSA, 0.2 mmol/L ATP), 3 ug/mL concatenated DNA (kinetoplast DNA (kDNA), a series or interlocking small rings of DNA; Profoldin, Hudson, MA), and various concentrations of DOX and GPX‐150 (100 µmol/L to 100 nmol/L) in half log increments or vehicle. The topoisomerase enzyme was added last to the reaction mixture to initiate the reaction. After 60 minutes, the reaction was stopped by addition of 5 mL stop solution (Profoldin, Hudson, MA) and the reaction was loaded onto a 96‐well filter plate (0.2‐µm PVDF membrane filter plate, Corning, Catalog #3504, Corning, NY) with an attached receiving plate. Plates were then centrifuged (4000 *g*) until all solution had passed through the filter. A quantity of 150 μL of rinse buffer (Profoldin, Hudson, MA) was loaded onto plates and centrifugation was repeated. Filter plate was then removed, and 50 μL of dye (Profoldin, Hudson, MA) was added to receiving plate. Each well was then excited at 485 nm and the intensity was read at 535 nm. Readings were then normalized to controls.

## 3 RESULTS

### 3.1 Patients characteristics

**Figure 1:**
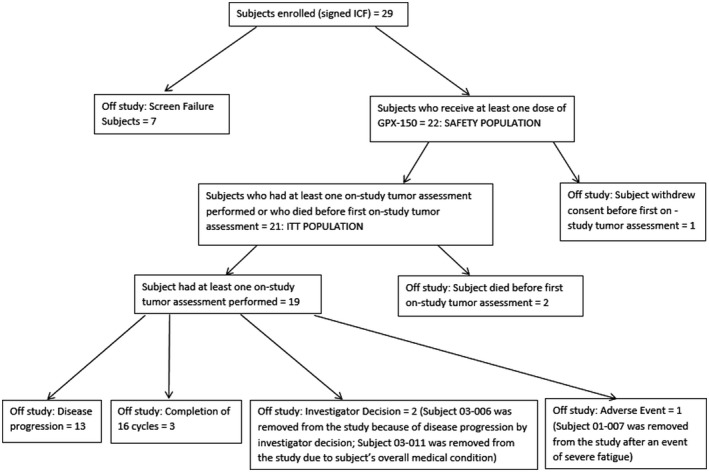
Disposition and accountability of patients

The patient demographics are shown in Figure 1 and Table 1. There were 22 patients in the safety population and 21 patients in the efficacy (intent to treat; ITT) population. Patients in the safety population received at least one dose of GPX‐150. Patients in the efficacy population received at least one tumor assessment after receiving a minimum of one dose of GPX‐150 although two of the 21 patients died before their first tumor assessment after receiving one dose of GPX‐150 (Figure 1).

**Table 1:** Patient characteristics

Characteristic	N	%
Safety population	22	
Efficacy population (intent to treat)	21	
Sex		
Male	15	68.2
Female	7	31.8
Age (y)		
Mean ± SD	59.4 ± 13.6	
Range	30‐84	
ECOG status		
ECOG 0	13	59
ECOG 1	9	41
ECOG 2	0	0
Race		
White	20	90.9
Black/African American	2	9.1
Identified Hispanic or Latino	2	9.1

The average age of patients in the safety population was 59.4 ± 13.6 years with a range of 30‐84 years. Thirteen patients were ECOG 0, 9 patients were ECOG 1, and no enrolled patients were ECOG 2. Fifteen subjects (68.2%) were male and seven (31.8%) were female. Twenty subjects (90.9%) were white and two subjects (9.1%) were Black/African American. 9.1% of subjects identified as Hispanic or Latino. The individual sarcoma histology classifications of the patients are shown in Table 2.

**Table 2:** Histology classification of the tumors at baseline (safety population)

	n = 22
Adipocytic tumor—myxoid/round cell liposarcoma	2 (9.1%)
Adipocytic tumor—pleomorphic liposarcoma	1 (4.5%)
Carcinosarcoma	1 (4.5%)
Dedifferentiated liposarcoma	3 (13.6%)
Fibrohistiocytic tumor—undifferentiated pleomorphic sarcoma/malignant fibrous	4 (18.2%)
Smooth muscle tumors (leiomyosarcoma)	5 (22.7%)
Tumor of peripheral nerves—malignant peripheral nerve sheath tumor	1 (4.5%)
Tumor of uncertain differentiation—synovial sarcoma	2 (9.1%)
Tumor of uncertain differentiation—undifferentiated sarcoma/sarcoma NOS	2 (9.1%)
Undifferentiated endometrial sarcoma	1 (4.5%)

### 3.2 Responses, PFS, and OS

**Figure 2:**
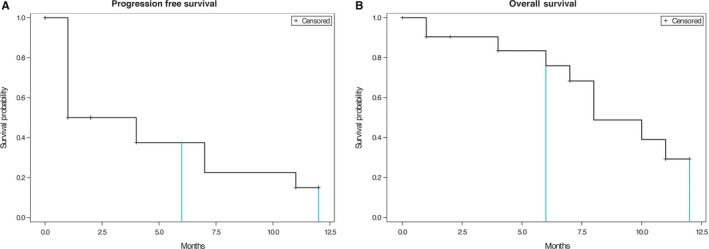
A. Kaplan‐Meier curve for progression free rate in STS patients receiving GPX‐150. B. Kaplan‐Meier curve for overall survival in STS patients receiving GPX‐150

The PFR of GPX‐150 at 6 and 12 months was 38% and 12%, respectively. The KM curve for PFS for GPX‐150 is shown in Figure 2A and OS in Figure 2B. The response rates were 0 Complete responses, 1, partial response and nine patients with stable disease. Patient response, time on treatment, number of doses, and cumulative dose for each patient are shown in Table 3.

**Table 3:** Extent of exposure to GPX‐150 (ITT population)

Subject no.	Total days on treatment	Doses received	Cumulative dose received (mg/m^2^)	Tumor response
01‐002	126	6	1590	SD
01‐003	18	1	265	Died
01‐004	42	2	530	PD
01‐005	47	2	530	PD
01‐007	140	6	1590	SD
03‐001	231	11	2915	SD
03‐002	32	2	465	Died
03‐003	371	16^a^	5300	SD
03‐004	48	2	530	PD
03‐006	42	2	530	PD
03‐007	239	10	2650	PR; SD
03‐008	42	2	530	PD
03‐010	56	2	530	PD
03‐011	86	4	1060	SD
03‐012	126	6	1590	SD
03‐013	365	16	4240	SD
04‐002	42	2	530	Died
04‐003	365	16	4240	SD
04‐004	58	2	530	PD
04‐005	43	2	530	PD
04‐006	44	2	530	PD

PD, progressive disease; PR, partial response; SD, stable disease.

a This subject continued dosing beyond the protocol‐specified number of doses and received a total of 20 doses of GPX‐150. This subject remained on study as a compassionate use subject for a total of 504 days on study. Median time on treatment was 56 days.

### 3.3 Safety

GPX‐150 was well tolerated in all patients including two patients who received 16 doses of GPX‐150 and one patient who received 20 doses of GPX‐150. Table 4 shows the percentage of patients with various side effects and severity of side effects for GPX‐150. GPX‐150 was not associated with significant alopecia or mucositis beyond grade 1 or with grade 3 or 4 nausea and vomiting.

**Table 4:** Percentage of patients with side effects and their grade for GPX‐150

Toxicity/side effect	GPX‐150
Infection	
Grade 3	0%
Grade 4	0%
Neutropenia/leukopenia	
Grade 3	0%
Grade 4	5%
Anemia	
Grade 2	9%
Grade 3	14%
Grade 4	0%
Febrile neutropenia	
Grade 3	5%
Grade 4	0%
Mucositis	
Grade 1	9%
Grade 2	0%
Grade 3	0%
Grade 4	0%
Alopecia	
Grade 1	9%
Grade 2	0%
Grade 3	0%
Nausea	
Grade 1	46%
Grade 2	14%
Grade 3	0%
Vomiting	
Grade 1	27%
Grade 2	9%
Grade 3	0%
Grade 4	0%

### 3.4 Cardiac safety

There was no overall trend for decreased LVEF as a function of cumulative dose in the safety population. The average LVEF at screening was 61.8 ± 6.5 (n = 22) and at the end of study was 58.4 ± 10.1 (n = 22) (not significant). The final visit mean LVEF was almost identical to the screening mean LVEF. There was a subset population of four subjects in which at least one abnormal LVEF below 50% occurred and could have been related to GPX‐150 administration. However, in all four subjects, all decreases in LVEF were transitory and returned to normal values and were not considered of clinical significance. The mean final LVEF (57.5 ± 2.6) in these four subjects was nearly identical to the mean screening LVEF (59.5 ± 2.6) and no subject developed clinical evidence of cardiotoxicity or heart failure.

As expected, no patient receiving GPX‐150 exhibited an abnormal ECG at any time during the study. The intervals were not prolonged or abnormal as a result of GPX‐150 administration and QTcF values were not different between screening and the final visit in the safety population.

### 3.5 Deaths during the study

Three patients died while on study. The first patient was diagnosed with high grade carcinosarcoma in 2015 and entered the GPX‐150 trial on 3 March 2015. While on vacation in Hawaii, he was hospitalized on 22 March 2015 for dehydration. Subsequent brain MRI showed four enhancing lesions in the right cerebellum and right occipital lobe. The investigator considered this event as disease progression after one GPX‐150 dose. The patient died from recurrent carcinosarcoma 9 days later on 31 March 2015. The second patient to die while on study had a medical history of leiomyosarcoma and received the first dose of GPX‐150 on 29 January 2015. On 3 February 2015, the patient developed febrile neutropenia, anemia, and decreased lymphocyte count likely related to GPX‐150 administration. The patient received a reduced dose of GPX‐150 per protocol on 19 February 2015. On 15 March 2015, the patient died at home from respiratory failure secondary to sarcoma not related to GPX‐150. This patient received doses of GPX‐150 and had been on study for 32 days.

The third patient to die while on study was a patient with a medical history of liposarcoma and received two doses of GPX‐150 (17 March 2015 and 7 April 2015). The patient died on 14 May 2015 from disease progression of liposarcoma as determined by the investigator.

### 3.6 Correlative analysis

To assess the relative potency of DOX and GPX‐150 to inhibit topoisomerase IIα and β‐mediated decatenation of DNA, concentrations of DOX and GPX‐150 ranging between 0.1 and 100 μmol/L were incubated with purified human topoisomerase Iiα sand IIβ and DNA at 37°C for 60 minutes. Table 5 shows the IC_50_ values for DOX and GPX‐150 to inhibit topoisomerase IIα and IIβ. Quantitation of decatenation by the fluorescence method shows that DOX is approximately 10 times more potent to inhibit topoisomerase IIα compared to IIβ. In contrast, GPX‐150 inhibited topoisomerase IIα but not IIβ. Thus, utilizing this methodology up to the concentration of 100 μmol/L, GPX‐150 was selective to inhibit topoisomerase IIα while DOX inhibited both topoisomerase IIα and IIβ isoforms.

**Table 5:** IC_50_ (μmol/L) of DOX and GPX‐150 to inhibit the in vitro decatenation of kDNA by human topoisomerase IIα and topoisomerase IIβ

	IC_50_: topoisomerase IIα	IC_50_: topoisomerase IIβ
DOX	3.8	40.1
GPX‐150	35.2	Not detectable

## 4 DISCUSSION

There has been an intense effort over the last five decades to remove the cardiotoxicity of DOX while retaining its anticancer activity or to develop cardioprotective strategies. The value of such an innovation is obvious with a decrease in the restriction in cumulative dosing and increasing the clinical utility in combination with other anticancer agents such as trastuzumab and paclitaxel.

Of the cardioprotective approaches, only dexrazoxane (Zinecard) has been approved by the FDA to attenuate DOX‐induced cardiotoxicity^23^ The mechanism of dexrazoxane cardioprotection is thought to relate to attenuated free radical formation via iron chelation and more recently it has been shown to be a catalytic inhibitor of topoisomerase IIβ, a mechanism recently implicated in the cardiotoxicity of DOX. However, its effect on cancer‐related outcomes remains controversial which has tempered FDA recommendations for use with DOX.^23^ For example, one study demonstrated a cardioprotective effect in women with breast cancer but the objective response of DOX was attenuated in combination with dexrazoxane vs placebo (ie, 46.8% vs 60.5%).^24^ Thus, the FDA has limited the use of dexrazoxane in patients with metastatic breast cancer who have received a cumulative dose of 300 mg/m^2^ or more of DOX.^23^

Another cardioprotective approach relates to how DOX is administered. Slow infusion of DOX appears to be less cardiotoxic that rapid infusion. In one study, a 6‐hour infusion of DOX was less cardiotoxic than a 20‐minute infusion.^25^ The mechanism may relate to decreased cardiac concentrations of DOX and the cardiotoxic metabolite, doxorubicinol.^25^

Other approaches have been directed to novel formulations such as PEGylated liposomal DOX,^26^ DOX conjugated to targeted moieties such as transferrin (Hofmann et al, 2007) or DOX linked to a protein such as albumin (aldoxorubicin).^27^, ^28^, ^29^, ^30^ GPX‐150 is a novel approach in that structural changes to the DOX molecule itself were utilized to reduce or eliminate the irreversible, cumulative dose‐dependent cardiotoxicity of DOX. In a preclinical study using a chronic rabbit model 20 and in a phase I clinical trial,^21^ GPX‐150 did not exhibit irreversible, cumulative dose‐dependent cardiotoxicity, even in patients previously treated with prior DOX or epirubicin. The results of this phase II study are consistent with the previous studies.

In a recent prospective study by Cardinale et al,^6^ 2625 DOX‐treated patients were followed for a median of 5.2 years after completion of DOX therapy. Two hundred and twenty‐six patients (9%) developed cardiotoxicity with a median time of 3.5 months after completion of DOX therapy. The investigators divided the cardiotoxic patients into three categories based on LVEF values; those who fully recovered to near pre‐DOX LVEF, those who partially recovered, and those who did not recover LVEF function. Only 11% of patients made a full recovery to pre‐DOX values and it required an average of 4 years for the mean LVEF to return to pre‐DOX values, even with treatment using ACE inhibitors and beta blockers. Thus, in this study, DOX cardiotoxicity was manifest within median of 3.5 months after completion of DOX therapy (approximately 9.5 months after start of DOX administration) and remained at abnormal values (<50%) for a year or longer in approximately 90% of patients with chronic, cumulative dose‐dependent DOX cardiotoxicity. These findings are also in agreement with another study which reported clinical heart failure within 1 month after completion of DOX.^31^

The Cardinale 6 and von Hoff 31 findings of DOX cardiotoxicity are in contrast to the results of the current phase II clinical trial with GPX‐150. Patients treated with GPX‐150 had normal mean LVEF values not statistically different from their pre‐GPX‐150 values (61.8 ± 6.5 at screening vs 58.4 ± 10.1, Table 5) 1 month or more after completion of GPX‐150 treatment that was up to 1 year after starting GPX‐150 treatment in three patients. In four patients, where a low (<50%) LVEF was recorded sometime during the study, the LVEF values 1 month or more after the final GPX‐150 dose was administered had returned to normal, pre‐GPX‐150 values (59.5 ± 2.6 at screening, 57.5 ± 2.6 at the end of the study, Table 1). Thus, cardiotoxicity was not an issue for GPX‐150 in this phase II study despite 16 or more doses (cycles) in three patients. In contrast, if DOX was administered at doses greater than 975 mg/m^2^, 55% of patients would be expected to develop congestive heart failure.^31^

If the cardiotoxicity of anthracyclines can be eliminated, the hope that continuous treatment with an anthracycline as opposed to a limited number of cycles would allow for improved PFS and OS. Contemporaneously with the development of GPX‐150, aldoxorubicin is another anthracycline that is being developed for the treatment of sarcoma that has reduced cardiotoxiticy. Chawla et al published a randomized phase IIb trial of aldoxorubicin vs DOX and found that aldoxorubicin had a superior PFS without evidence of cardiotoxicity when compared to DOX.^29^ In this study, treatments were limited to six cycles for both drugs. This demonstrates the efficacy of increased exposure of STS to anthracyclines. Given that this trial was likely performed with GPX‐150 at a dose that is below a true maximum tolerated dose, it will take subsequent trials to determine whether aldoxorubicin or GPX‐150 will become the lead anthracycline replacement of choice. Until then, further investigation into both compounds is needed.

The sample size in this study is too small to draw conclusions about histologies associated with antitumor response or the transient fall in LVEF in the four patients in this study. Other factors besides GPX‐150 may have been responsible for a low LVEF recording. Nevertheless, these decreases in LVEF in GPX‐150–treated patients were not serious adverse events and were transient, with LVEF subsequently returning to normal levels in all four subjects. Despite some subjects receiving GPX‐150 for up to 16 cycles, effects on cardiac function were of no clinical significance and there was no evidence of irreversible heart failure in any subject. In all instances, decreases in LVEF were transitory and self‐limited.

In this study, GPX‐150 demonstrated limited toxicity as seen from its safety profile. Apart from one patient who developed febrile neutropenia and severe leukopenia, there were no grade 4 toxicities reported and no grade 3 side effects apart from anemia (Table 4). Indeed, the toxicity profile of GPX‐150 was better than with DOX as illustrated by a 2% occurrence of grade 4 and 6% occurrence of grade 3 mucositis with DOX^32^ but there were no grade 3 or 4 mucositis toxicities observed with GPX‐150 in the current study (Table 4). Similarly, DOX caused a 21% grade 3 alopecia rate in one study^33^ but there were no grade 3 or 4 GPX‐150 patients with alopecia (Table 4). Doxorubicin also caused 13% of patients to elicit grades 3 and 4 nausea and vomiting^32^ while no GPX‐150–treated patient had grade 3 or 4 nausea or vomiting. This suggests that doses higher than 265 mg/m^2^ could be used in future studies possibly with even better efficacy outcomes. The dose chosen in the current study was based on the phase I clinical study in which GPX‐150 was administered in the absence of G‐CSF, which is not truly a maximum tolerated dose of this therapeutic. In the original phase I, due to the lack of G‐CSF, 5 of 7 patients at 265 mg/m^2^ required dose lowering due to neutropenia, and this was called the MTD. In the current phase II study, G‐CSF was administered prophylactically and only one patient exhibited a neutropenia. Since neutropenia is not the dose limiting toxicity for GPX‐150 in the presence of G‐CSF, higher doses of GPX‐150 should be explored in STS and evaluated for better clinical efficacy outcomes.

The clinical benefit of GPX‐150 in the patient with STS must be conservatively examined due to the very small sample size in this study. First, two patients died rapidly thought they were thought by investigators to have at least a 3‐month OS at the time of study entry. If patients have chemorefractory rapidly progressive disease, as can happen with sarcoma, this can happen. This effect was also seen with the early deaths in the DOX arm of the Olaratumab trial.^5^ Second, when one examines the PFS at 6 months, we find a 38% clinical benefit rate which is equivalent to the control arm of the olaratumab trial in terms of benefit.^5^ In addition, there were two patients that were on this therapy for the planned 16 cycles without substantial toxicity. In a small trial, this is a sign of clinical activity and benefit to the patients that received therapy. Finally, whether the lack of response rate (only one patient) is due to sample size, GPX‐150 dose, or another reason will have to await formal testing in a larger trial.

In conclusion, the results from this phase II clinical study indicate that GPX‐150 is better tolerated and has a better safety profile than DOX. Future studies will help to further define the clinical role of GPX‐150 in STS and other cancers.

## CONFLICT OF INTERESTS

Brian A. Van Tine served in a consulting or advisory role for Novartis, Lilly/ImClone, Johnson & Johnson, Caris Life Sciences, DFINE, Threshold Pharmaceuticals, Karyopharm Therapeutics. Speaker's Bureau: Caris Life Sciences, Jansen, and Lilly. Mark Agulnik served in a consulting or advisory role for: EMD Serono, Janssen Pharmaceuticals, Eisai, Novartis. Speaker's Bureau: Novartis, GlaxoSmithKline, Janseen and Lilly. Mohammed Milhem served in a consulting or advisory role: EMD Serono, Amgen, Eisai, Novartis, Genentech, BMS. Richard D. Olson served in a consulting or advisory role for Gem Pharmaceuticals, LLC. Gerald M. Walsh served in a consulting or advisory role for Gem Pharmaceuticals, LLC. Arthur Klausner served in a consulting or advisory role for Gem Pharmaceuticals, LLC. All the remaining authors have declared no conflicts of interest.
